# Solar ultraviolet radiation is necessary to enhance grapevine fruit ripening transcriptional and phenolic responses

**DOI:** 10.1186/1471-2229-14-183

**Published:** 2014-07-09

**Authors:** Pablo Carbonell-Bejerano, Maria-Paz Diago, Javier Martínez-Abaigar, José M Martínez-Zapater, Javier Tardáguila, Encarnación Núñez-Olivera

**Affiliations:** 1 Instituto de Ciencias de la Vid y del Vino (ICVV), Consejo Superior de Investigaciones Científicas-Universidad de La Rioja-Gobierno de La Rioja, Madre de Dios 51, 26006 Logroño, Spain; 2 Universidad de La Rioja, Edificio Científico-Tecnológico, Madre de Dios 51, 26006 Logroño, Spain

**Keywords:** Anthocyanins, Flavonols, Fruit ripening, Grapevine, Microarray, Phenolic compounds, Stilbenes, Terpenoids, Ultraviolet radiation, *Vitis vinifera*

## Abstract

**Background:**

Ultraviolet (UV) radiation modulates secondary metabolism in the skin of *Vitis vinifera* L. berries, which affects the final composition of both grapes and wines. The expression of several phenylpropanoid biosynthesis-related genes is regulated by UV radiation in grape berries. However, the complete portion of transcriptome and ripening processes influenced by solar UV radiation in grapes remains unknown.

**Results:**

Whole genome arrays were used to identify the berry skin transcriptome modulated by the UV radiation received naturally in a mid-altitude Tempranillo vineyard. UV radiation-blocking and transmitting filters were used to generate the experimental conditions. The expression of 121 genes was significantly altered by solar UV radiation. Functional enrichment analysis of altered transcripts mainly pointed out that secondary metabolism-related transcripts were induced by UV radiation including *VvFLS1*, *VvGT5* and *VvGT6* flavonol biosynthetic genes and monoterpenoid biosynthetic genes. Berry skin phenolic composition was also analysed to search for correlation with gene expression changes and UV-increased flavonols accumulation was the most evident impact. Among regulatory genes, novel UV radiation-responsive transcription factors including *VvMYB24* and three bHLH, together with known grapevine UV-responsive genes such as *VvMYBF1*, were identified. A transcriptomic meta-analysis revealed that genes up-regulated by UV radiation in the berry skin were also enriched in homologs of Arabidopsis *UVR8* UV-B photoreceptor-dependent UV-B -responsive genes. Indeed, a search of the grapevine reference genomic sequence identified UV-B signalling pathway homologs and among them, *VvHY5-1*, *VvHY5-2* and *VvRUP* were up-regulated by UV radiation in the berry skin.

**Conclusions:**

Results suggest that the UV-B radiation-specific signalling pathway is activated in the skin of grapes grown at mid-altitudes. The biosynthesis and accumulation of secondary metabolites, which are appreciated in winemaking and potentially confer cross-tolerance, were almost specifically triggered. This draws attention to viticultural practices that increase solar UV radiation on vineyards as they may improve grape features.

## Background

Cultivated grapevines are normally exposed to UV radiation reaching the Earth’s surface (8-9% of the total amount of solar radiation). Only UV-A radiation (wavelengths between 315–400 nm, 6.3%) and UV-B radiation (280–315 nm, 1.5%) reach the ground; principally because UV-C radiation (<280 nm), which is extremely harmful, is absorbed by stratospheric oxygen, ozone and other atmospheric gases [1]. The UV irradiance reaching the Earth’s surface increases with altitude and decreases with latitude [2]. In viticulture, UV irradiance reaching the plants also depends on the vineyard orientation and slope, as well as on environmental features such as cloudiness, etc. [3]. Grapevine is generally adapted to environmental UV radiation doses, which are not stressing for the physiology of the vines [3-5]. Rather, solar UV radiation represents an environmental signal modulating physiological characteristics of vines including the accumulation of secondary metabolites in the skin of ripening berries [6-11]. Therefore, the impact of UV radiation on the vines and the dose received can be relevant variables to be considered by winegrowers.

Plants are necessarily exposed to solar UV radiation because they require sunlight to carry out photosynthesis. They are generally adapted to environmental UV-B radiation exposure since they have evolved mechanisms to avoid being damaged. Protective barriers comprise the accumulation of UV radiation-absorbing compounds (mainly phenolics) in epidermal and subepidermal cell layers to limit the incidence of UV radiation over inner layers [12,13]. Additionally, mechanisms to restore injuries provoked by the action of UV radiation have also been developed including different DNA repair mechanisms and antioxidant systems [14-16]. Although some of these defences are constitutively present, they can also be enhanced under increased UV radiation [13,17]. Besides photoprotection, UV radiation also triggers safeguards to anticipate other stressors such as heat or drought, and mediates in developmental cues such as morphogenetic responses to shade/light or interactions with other organisms [18-22]. Indeed, a sensing and signalling pathway that specifically perceives UV-B radiation has been discovered in *Arabidopsis thaliana* L. involving a regulatory cascade initiated at the UVB-RESISTANCE 8 (UVR8) UV-B radiation photoreceptor, which controls gene expression to trigger morphogenetic, metabolic, protective and repair mechanisms [22-24].

Fruits and seeds are vital plant organs to ensure species propagation and, as such, protective mechanisms can be important to guarantee proper embryo development and seed dispersal. Flavonoids are chief compounds in photoprotection not only because of their UV radiation-screening capacity, but because they are presumably involved in other functions such as counteracting high light-induced oxidative damage [25]. Flavonoids accumulate in the berry skin, which includes an epidermal and several hypodermal cell layers [26-28]. Accumulation of anthocyanins, flavonols and other phenolic compounds in the grape berry skin is strengthened from the inception of ripening (veraison) and their concentration can be increased when grapes are exposed to sunlight [10,29-31]. Flavonols are flavonoids that have the 3-hydroxyflavone backbone whose accumulation in the berry skin is greatly enhanced in the presence of UV radiation and indeed, their content has been related to the grape skin UV-A radiation-absorbing capacity [6,10,27]. There is also evidence indicating flavonols involvement in plant antioxidant and signalling activities [25]. The content of non-flavonoid hydroxycinnamic acids is more correlated with the berry UV-B radiation-absorbing capacity although, similarly to flavanols content, they do not clearly increase in grapes in response to UV radiation [7,11,27,32]. Anthocyanin pigments accumulation also increases in the grape skin of black-skinned cultivars as a consequence of UV radiation; although high UV irradiances such as those received at high altitudes seem to be required for triggering the response [7,33-35]. Mainly photoprotective and antioxidant functions are proposed for UV radiation-responsive anthocyanins according to their weak UV radiation-absorption capacity; although acylation reactions convert them in better UV-screeners [16,36,37]. Accumulation of stilbenes and volatile compounds in the skin of Malbec grapes is also enhanced by the UV received at high altitudes [7,8]. UV radiation-induced compounds are appreciated for different uses of grapes because they improve berry and wine features such as aroma, astringency, colour and stability; while they can also increase grapes tolerance to abiotic and biotic stressors [3].

Concurrently to changes in the grapevine berry biochemical composition, UV radiation up-regulates the expression of genes encoding enzymes involved in the biosynthesis of flavonoids and their precursors [11,34]. Expression of genes leading to flavonols production in the berry skin is usually more highly induced by UV radiation than those of other phenylpropanoid biosynthetic and pathway regulatory genes [11]. Nonetheless, the proportion of secondary metabolism-related genes or the signalling pathways that are activated by the effect of UV radiation on ripening grapes still remains unknown; since its impact on the grape transcriptome has not been globally analysed.

The goal of this study is to characterize the transcriptome that is affected by solar UV radiation on the berry skin of grapes grown at mid-altitude and how the phenolic composition is altered by it. The presence of the UV-B signalling pathway in grapevine and its activation in the skin of berries exposed to the environmental UV radiation are also explored.

## Methods

### Plant material and experimental design

The field experiment was conducted in the 2012 season in a commercial vineyard located in Mendavia (Navarra, northern Spain, 42° 27’ N, 2° 14’ W, 371 m asl). *Vitis vinifera* L. cv. Tempranillo, grafted onto 110R rootstocks and planted in 2007 on clay-loam soil with NE-SW row orientation, was used. The vines were spur-pruned (12 buds per vine) in a bilateral cordon and trained to a VSP (vertical shoot positioning) trellis system. At pre-bloom (7 June 2012, seven days before flowering), vines were partially defoliated by removing the first six main basal leaves to increase and homogenize the exposure of fruits to solar radiation. Shoots were trimmed once at the end of July, before veraison. Vines were not irrigated during the growing season.

A completely randomized block design was set-up. Three blocks of nine vines were divided into three experimental conditions (three vines per replicate): no filter (Ambient); UV radiation-transmitting filter (FUV+); UV radiation-blocking filter (FUV-). The two filtered treatments were established using colourless and transparent polymetacrylate filters (PMMA XT Vitroflex 295 and XT Vitroflex 395 Solarium Incoloro, Polimertecnic, Girona, Spain), which allowed for and blocked, respectively, the transmission of UV radiation. Filters (1.30 × 0.75 m) were placed at 45° from the vertical axis of the plant, on both sides of the canopy, covering the fruiting zone and the first 0.7 m of the canopy of each grapevine. Filters were installed right after defoliation and maintained until harvest (7 September 2012). Spectral irradiances below filters were measured regularly from the beginning of the experiment using a spectroradiometer (Macam SR9910, Macam Photometrics Ltd, Livingstone, Scotland) to confirm the stability of their filtering characteristics. Environmental photosynthetic (PAR), UV-A, and UV-B radiations were continuously recorded close to the experimental plot with broad band radiometers (Skye Quantum SKP 215, SKU 420 and SKU 430, respectively, Skye Instruments Ltd, Powys, UK) installed at Universidad de La Rioja. The biologically effective UV irradiance (UV_BE_) was estimated using the action spectrum of Flint and Caldwell [38].

At veraison (1 August 2012), fruit temperatures were determined by thermography in each replicate to check the influence of filters. Thermal images were taken at solar noon with a thermal camera (ThermaCAM P640, FLIR Systems, Sweden) as in Pou et al. [39].

### Berry sampling

For all treatments, berry samples near commercial maturity were collected around noon on a sunny day (7 September 2012). Nine clusters were collected for each replicate (three clusters per plant), always from the basal position of a SE orientated shoot. Every berry was separated from its cluster by cutting the pedicel and its density was determined by floatability in a NaCl solution series as a non-invasive indication of the internal sugar concentration [40,41]. This sampling method allowed for harvesting simultaneously and from the same clusters berries at different known ripening states. This was done to avoid environmental differences other than the UV radiation that could influence on gene expression. For each replicate, all berries on every density interval were weighed together to calculate relative berry abundance. Total soluble solids (TSS) of berries in each density interval were measured by a digital refractometer WM-7 (ATAGO, Tokyo, Japan). Berries with density between 130–150 and between 160–180 g l-1 NaCl (corresponding to TSS of approximately 23 and 26 ºBrix, respectively) were rinsed in distilled water, immediately frozen in liquid nitrogen and kept at -80°C until further analyses.

### Analysis of phenolic compounds

Frozen berries (-80°C) were allowed to partially thaw and skin was carefully removed from the flesh using a scalpel without rupturing the hypodermal cells. The skins were immediately submerged in liquid nitrogen, weighed and grounded for 20 s with an analytical mill (A11 basic, IKA, Staufen, Germany) until a very fine paste was obtained. For each analytical sample, 50 mg of the paste were frozen in liquid nitrogen and ground in a TissueLyser (Qiagen, Hilden, Germany). Then, five ml of methanol: water: 7 M HCl (70:29:1 v:v:v) was added for extraction (24 h at 4°C in the dark). The extract was centrifuged at 6000 *g* for 15 min and the supernatant and pellet were considered the source of, respectively, methanol-soluble and methanol-insoluble phenolic compounds (MSPC and MIPC, respectively). Soluble compounds are mainly located in the vacuoles whereas insoluble compounds are bound to the cell walls [42]. In both fractions, the bulk level and the concentrations of different individual phenolic compounds were measured. Bulk levels of MSPC and MIPC per unit of fresh weight (FW) were measured as in Fabón et al. [43]. Individual phenolic compounds were measured either by HPLC (anthocyanins) or UPLC-MS (non-anthocyanins). HPLC determinations (Agilent HP1100 HPLC system, Agilent Technologies, Palo Alto, CA, USA) followed Gómez-Alonso et al. [44]. UPLC analyses were carried out using the Waters Acquity Ultra Performance LC system (Waters Corporation, Milford, USA) following Saenz-Navajas et al. [45] with modifications. Solvents were: A, water/formic acid (0.1%); and B, acetonitrile with 0.1% formic acid. The gradient program employed was: 0–7 min, 99.5-80% A; 7–9 min, 80-50% A; 9–11.7 min, 50-0% A; 11.7-15 min, 0–99.5% A. The UPLC system was coupled to a micrOTOF II high-resolution mass spectrometer (Bruker Daltonik, Germany) equipped with an Apollo II ESI/APCI multimode source and controlled by the Bruker Daltonics DataAnalysis software. The electrospray source was operated in negative mode. The capillary potential was set to 4 kV; the drying gas temperature was 200°C and its flow 9 l · min^-1^; the nebulizer gas was set to 3.5 bar and 25°C. Spectra were acquired between *m/z* 120 and 1505 in negative mode. The different phenolic compounds analysed were identified according to their order of elution and retention times for pure compounds: catechin, epicatechin, catechin gallate, epicatechin gallate, myricetin, quercetin, caffeic acid, coumaric acid, ferulic acid and *t*-resveratrol (Sigma, St. Louis, USA); malvidin-3-glucoside, procyanidin B1, quercetin, kaempferol, isorhamnetin glucoside, and kaempferol-3-rutinoside (Extrasynthese, Genay, France); quercetin-3-rutinoside, isorhamnetin and quercetin-3-galactoside (Fluka, Buchs, Germany). Quantification of non-commercial compounds was carried out using the calibration curves belonging to the most similar compound: malvidin-3-glucoside for the anthocyanins; quercetin-3-glucoside for quercetin; caffeic acid for caftaric acid; *p*-coumaric acid for coutaric acid; and *t*-resveratrol for its glucoside. Total amount of anthocyanins was given in mg · g^-1^ FW (skin) of malvidin-3-glucoside because it was the only standard used for quantification of anthocyanins; whereas total amounts of flavonols and hydroxycinnamic acid derivatives were expressed in μg · g^-1^ FW (skin) because several standards were used for quantification.

### Statistical analysis of phenolic composition

The effects of treatment and berry density on phenolic composition were tested using a two-way analysis of variance (ANOVA), once known that the data met the assumptions of normality (Shapiro–Wilk test) and homoscedasticity (Levene’s test). In the case of significant differences, means were compared by the Tukey’s test. Non-parametric tests (Kruskal-Wallis) were used if the data did not meet the assumptions. In this case and, when significant differences occurred, means were compared by the Mann-Whitney’s test. When only two set of data had to be analysed, differences between them were assessed using the Student’s *t* tests. All the statistical procedures were performed utilising the SPSS 19.0 software for Windows (SPSS Inc., Chicago, USA).

### Gene expression analyses

#### RNA isolation

Frozen berries were peeled and total RNA was extracted from frozen berry skin as described by Reid et al. [46]. DNase digestion of contaminating DNA in the RNA samples was carried out with the RNase-Free DNase Set (QIAGEN). Final RNA purification was carried out using the Spektrum™ Plant Total RNA kit (Sigma-Aldrich) according to standard protocols.

#### Microarray hybridization and data processing

RNA integrity for each RNA preparation was tested using an Agilent 2100 Bioanalyzer (Agilent technologies). cDNA was synthesized from 10 μg of total RNA using the cDNA Synthesis System Kit (NimbleGen-Roche). The cDNA preparation (1 μg) was amplified and labelled with Cy3-random nonamers using the One-Color Labelling Kit (NimbleGen-Roche). If the bioanalyzer quality control was correct, then 4 μg of labelled cDNA were hybridized on a NimbleGen microarray 090818 Vitis exp HX12 (NimbleGen-Roche). Hybridization solution (NimbleGen Hybridization kit) was added to each labelled cDNA and hybridization was performed for 16 h at 42°C in a HS 4 Hybridization station (NimbleGen-Roche). Hybridized microarrays were washed with the Wash buffer kit (NimbleGen-Roche) and scanned at 532 nm and 2 μm resolution in a DNA Microarray Scanner with the Surescan High-Resolution Technology (Agilent technologies).

After evaluation of hybridization quality by the experimental metrics report implemented in the NimbleScan Software version 2.6 (NimbleGen-Roche), probeset signal values from all microarray hybridizations were background corrected and normalized together using the robust microarray average (RMA) [47] with the NimbleScan Software as well, which produces calls file for each sample with normalized expression data condensed for each gene. A dataset was generated from normalized data including the expression of all 29,549 genes represented in the microarray in the 12 analysed samples (Additional file 1). A principal component analysis (PCA) [48] was directed over this dataset on the Qlucore Omics Explorer version 2.3 (Lund, Sweden).

#### Identification of differentially expressed transcripts and functional analysis

Berry skin RNA from FUV+ and FUV- was compared in the NimbleGen microarrays as the most suitable comparison to specifically analyse the effect of UV radiation on gene expression, minimizing other possible filter screen consequences such as concentration of heat or differences to wind exposure. A two-factor ANOVA analysis (Factor A: UV irradiation treatment; Factor B: berry density) was conducted in MeV [49] to detect differential expression produced by UV irradiation incidence on the skin of ripening berries and/or its interaction with the ripening degree. Transcripts differentially expressed (DE) by the effect of solar UV radiation were selected according to a *P* ≤0.01 for UV radiation factor or for the interaction UV radiation × density factors and FUV+/FUV- fold change ≥2 in at least one berry density. Transcripts with *P* ≤0.01 for density factor and fold change ≥2 between both densities were considered as density-DE.

K-means with Euclidean squared metrics and scaled rows also run in Acuity 4.0 (Axon Molecular Devices, http://www.moleculardevices.com) was used for clustering of UV-DE transcripts according to their mean Log_2_ (FUV+/FUV-) expression ratio on both analysed berry densities. Three clusters were generated as assessed in a Gap statistical analysis [50] run also in Acuity 4.0. A heat-map showing in all 12 samples the row normalized expression of UV-DE transcripts grouped in the three k-means resulting clusters was produced on the Qlucore Omics Explorer version 2.3. UV-DE transcripts from each cluster as well as density up- and down-regulated transcripts were analysed on Babelomics suite [51] to search for significant functional enrichment following a grapevine specific functional classification of 12X V1 predicted transcripts [52]. The Fisher’s exact test was used in a FatiGO analysis [53] to compare each study list to the list of total transcripts housed in the grapevine 12X V1 gene predictions [52]. Significant enrichment was considered in case of *P* ≤0.05 after the Benjamini and Hochberg correction. Using the same criteria, enrichment within each cluster was analysed for homologs of UVR8-dependent UV-B-induced genes in Arabidopsis leaves [54]. To this end, the best Arabidopsis match for each grapevine transcript in the NimbleGen microarray was considered as published in Grimplet et al. [52]. Redundancy in Arabidopsis homologs was summarized on each analysed list and finally, enrichment in 55 UVR8-dependent homologous genes from the 11,673 Arabidopsis homologs represented in the grapevine NimbleGen microarray and present in the Affymetrix ATH1 microarray was studied for each cluster.

### Search of UV signalling gene homologs

The grapevine genomic sequence was searched for loci encoding homologous proteins to Arabidopsis UVR8, HY5, COP1, RUP1 and RUP2 UV-B signalling components. For each Arabidopsis protein sequence, a BLAT alignment against the grapevine reference genomic sequence (PN40024 12X version) was carried out in the Genoscope website (http://www.genoscope.cns.fr/blat-server/cgi-bin/vitis/webBlat) to search for their grapevine homologs. For each locus, the corresponding 12X V1 version protein ID was identified from Grimplet et al. [52]. Grapevine 12X V1 protein sequences were obtained from the Uniprot website (http://www.uniprot.org/) and were aligned to Arabidopsis protein sequences by blastp (http://blast.ncbi.nlm.nih.gov/) to analyse the similarity.

## Results

### Experimental conditions of radiation and temperature

**Figure 1:**
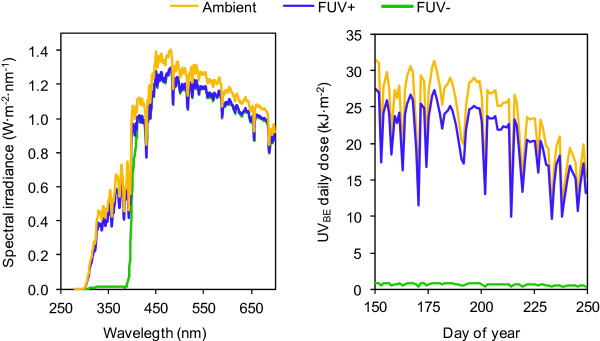
**Radiation received by plants under each treatment.** Left, spectral irradiances measured in the three treatments used: no filter (Ambient), UV-transmitting filter (FUV+), and UV-blocking filter (FUV-). Right, daily doses of biologically effective UV radiation (UV_BE_) received by the plants during the experiment (7 June to 7 September 2012) in the three treatments.

As a first approach in defining differential conditions, which could be generated between treatments, radiation and temperature parameters were evaluated in all three assayed settings. UV radiation was almost absent under FUV-; while FUV+ only produced a slight irradiance reduction compared to the control (Ambient) situation (Figure 1). UV_BE_ doses received by Tempranillo experimental vines from the onset of treatments until harvest time were 51, 1524 and 1782 kJ · m^-2^ for FUV-, FUV+ and Ambient treatments, respectively (Figure 1B). During that period, ambient UV-A and UV-B daily radiation doses varied between 524–1139 and 13–32 kJ m^-2^, respectively. Fruit temperature, measured around solar noon time at veraison, was only significantly different (*P* = 0.012) under FUV+ although the mean difference was less than 1°C: 31.35 ± 0.65°C (mean ± SD) in FUV+ when compared to 30.96 ± 0.54°C and 30.49 ± 0.45°C in FUV- and Ambient treatments, respectively.

### Effect of radiation treatments on berry development and ripening

**Figure 2:**
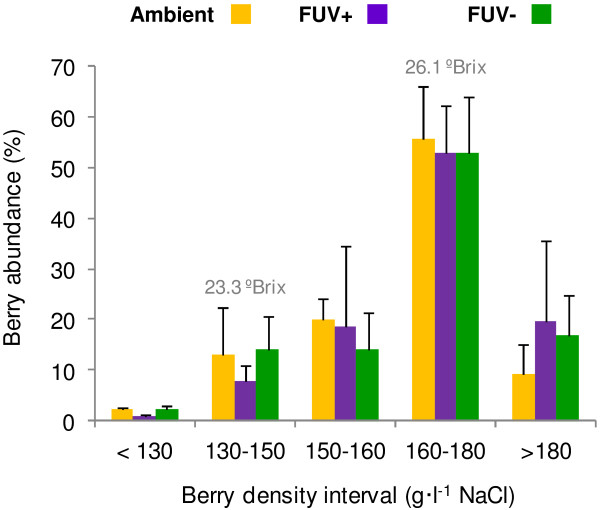
**Effect of radiation treatments on berry ripening at harvest time.** Berry density was determined by floatation in a NaCl solution series for each treatment: Orange, Ambient (no filter); Purple, UV-transmitting filter (FUV+); Green, UV-blocking filter. Berry TSS (ºBrix) on each density interval were measured by a refractometer and mean values are shown above the bars of the harvested intervals. Data are means from three blocks per treatment. Black bars represent SD. Berry density distribution differences between treatments were not significant for any berry density interval (*P* >0.05 in every two-way ANOVA).

The three treatments did not generate differences in berry saccharimetric ripening given the similar distribution of berry density abundance observed at harvest. In all three treatments, a majority of berries had a density between 160–180 g · l^-1^ NaCl corresponding to TSS of 26.1 ± 0.8 ºBrix (Figure 2). Berry weight and skin to berry ratio were not considerably affected by the treatments (Additional file 2). These parameters were also comparable between berries of 130–150 g · l^-1^ NaCl (23.3 ± 0.9 ºBrix) and 160–180 g · l^-1^ NaCl ripening stages; except for berry weight under FUV+, which was slightly lower in ~23 ºBrix berries (*P* = 0.037).

### Effect of radiation treatments on the phenolic composition of Tempranillo berry skin

**Figure 3:**
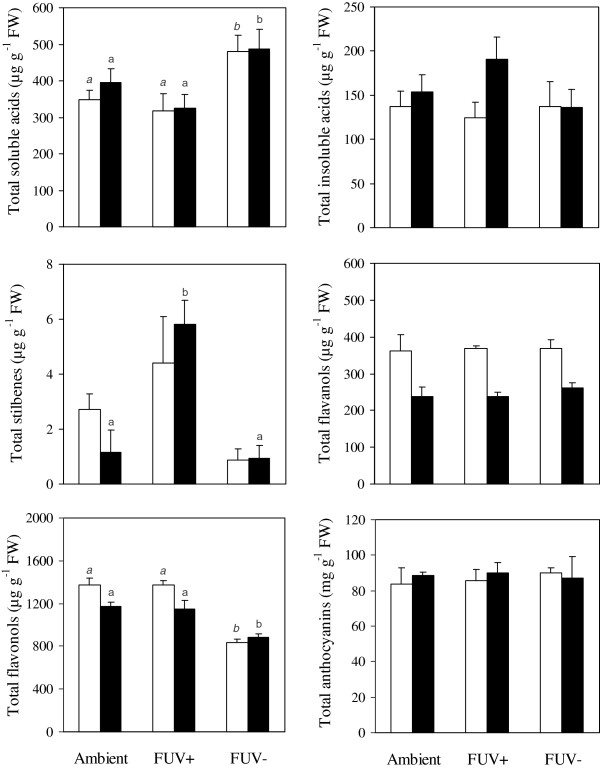
**Effects of radiation treatment and berry ripening on the accumulation of phenolic compounds.** Levels of measured compounds grouped in families are shown. Treatments were: no filter (Ambient), UV-transmitting filter (FUV+) and UV-blocking filter (FUV-) and berry ripening levels corresponded to 23 ºBrix (white bars) and 26 ºBrix (black bars). The compounds analysed were grouped in phenolic acids from the methanol-soluble and -insoluble fractions, stilbenes, flavanols, flavonols and anthocyanins. Means ± SE are shown. Different letters indicate significant differences (at least at *P* <0.05) between treatments for the 23 ºBrix (italics) and 26 ºBrix (normal type) berries.

The berry skin phenolic composition was analysed to test the effect of the UV radiation dose received in a mid-altitude vineyard and to compare it with its effect on the transcriptome. Total levels of MSPC and MIPC present in the berry skin were hardly affected by the radiation conditions (Table 1). Nonetheless, radiation treatments displayed significant effects on the levels of some phenolic compound families as observed when individual compounds were grouped according to these (Figure 3). Flavonols content was higher whereas phenolic acid levels from the methanol-soluble fraction were lower in the presence of UV (Ambient and FUV+). Stilbene levels were higher in FUV+; while phenolic acids from the methanol-insoluble fraction, flavanols, and anthocyanins did not show any significant variation. Accordingly to the affected families, 22 out of the 41 individual phenolic compounds analysed showed significant differences between treatments. Levels of one hydroxybenzoic acid (protocatechuic), one hydroxycinnamic acid (*p*-coumaric) and nine flavonols were significantly higher in both treatments that received solar UV radiation (Ambient and FUV+) than in the one deprived of it (FUV-). Among them, only *p*-coumaric acid was found in the methanol-insoluble fraction. All nine UV radiation-increased flavonols were glycosylated (two kaempferols, four quercetins and three isorhamnetins). They included all detected flavonol hydroxylation forms with the exception of the trisubstituted forms (myricetin and its 3′,5′-dimethoxyl derivative syringetin). Glucosylated forms of quercetin and the *cis* glucosylated isomer of isorhamnetin were not UV-responsive. UV radiation also increased the levels of petunidin-3-*O*-(6´-acetyl) glucoside and delphinidin-3-*O*-(6´-*p*-coumaroyl) glucoside anthocyanins; although differences were significant only in 26 ºBrix berries. Coumaroyl-tartaric acid was the only compound whose levels fell in presence of solar UV radiation in both analysed berry ripening stages. Concerning stilbenes, an UV radiation-inductive effect was observed for *trans*-piceid in 23 ºBrix berries. In contrast, *trans*-piceid in 26 ºBrix as well as resveratrol levels in both analysed berry densities were higher in FUV+ when compared to the other two treatments.

**Table 1:** Effects of radiation treatment (Ambient, no filter; FUV+, UV-transmitting filter; FUV-, UV-blocking filter) and berry saccharimetric level on the phenolic composition of skins in Tempranillo berries

**Phenolic compounds**	**23 ºBrix Ambient**	**23 ºBrix FUV+**	**23 ºBrix FUV-**	**26 ºBrix Ambient**	**26 ºBrix FUV+**	**26 ºBrix FUV-**	***P*-rad**	***P*-s**
MSPC	15.0 ± 2.0	15.8 ± 0.8	16.8 ± 0.9	15.6 ± 0.9	15.5 ± 0.7	15.1 ± 1.7	0.632	0.799
MIPC	9.4 ± 0.1	10.4 ± 0.5	11.8 ± 1.2	9.6 ± 0.7	8.2 ± 1.4	10.3 ± 2.3	0.031	0.587
**Phenolic acids (μg · g**^**-1**^**FW)**								
Protocatechuic acid	7.7 ± 0.3a	5.5 ± 0.2b	1.3 ± 0.3c	5.6 ± 0.0a	3.1 ± 0.4a	0.9 ± 0.1b	0.000	0.000
Caffeoyl-tartaric acid	69.7 ± 9.5	78.7 ± 34.3	115.1 ± 8.4	145.1 ± 22.7	117.7 ± 5.3	181.2 ± 14.8	0.063	0.015
Coumaroyl-tartaric acid	271.7 ± 24.6a	232.7 ± 19.5a	364.3 ± 26.4b	244.7 ± 20.2a	204.3 ± 25.2a	306.0 ± 35.4a	0.002	0.997
*p*-coumaric acid	111.4 ± 4.5a	87.4 ± 1.0ab	75.1 ± 4.5b	111.0 ± 0.8a	128.7 ± 14.6a	79.9 ± 8.1b	0.003	0.028
Syringic acid	26.1 ± 7.1	36.7 ± 12.7	61.8 ± 25.5	42.2 ± 8.7	62.2 ± 12.7	56.3 ± 2.2	0.253	0.236
**Stilbenes (μg · g**^**-1**^**FW)**								
Resveratrol	0.4 ± 0.0a	1.1 ± 0.0b	0.3 ± 0.1a	0.4 ± 0.2a	1.2 ± 0.0b	0.3 ± 0.2a	0.035	0.407
*Trans*-piceid (resveratrol-3-*O*-glucoside)	2.4 ± 0.5a	3.3 ± 0.2a	0.6 ± 0.2b	0.8 ± 0.4a	4.6 ± 0.7b	0.6 ± 0.3a	0.004	0.351
**Flavanols (μg g**^**-1**^**FW)**								
Catechin	48.3 ± 10.0	63.3 ± 5.3	54.6 ± 3.1	30.6 ± 3.3	34.1 ± 3.5	44.8 ± 6.9	0.165	0.001
Epicatechin	9.6 ± 1.3a	11.1 ± 0.5a	8.2 ± 0.6a	5.3 ± 0.4a	8.4 ± 1.0b	7.1 ± 0.3b	0.030	0.002
*Cis*-epigallocatechin	129.4 ± 10.6a	96.1 ± 4.1b	133.9 ± 11.5a	86.7 ± 2.6a	81.8 ± 4.7a	91.5 ± 5.6a	0.016	0.000
*Trans*-epigallocatechin	35.0 ± 3.0	27.1 ± 1.1	26.7 ± 0.5	24.6 ± 0.1	24.9 ± 2.1	27.0 ± 5.8	0.514	0.166
Procyanidin B1	138.6 ± 7.8	142.6 ± 5.2	145.2 ± 8.2	89.8 ± 0.2	88.9 ± 4.4	90.7 ± 5.8	0.851	0.000
**Flavonols (μg · g**^**-1**^**FW)**								
Myricetin	86.8 ± 1.1a	58.6 ± 2.6b	45.8 ± 0.3c	79.0 ± 8.7a	56.8 ± 1.2a	45.8 ± 7.5a	0.004	0.497
Myricetin-3-*O*-glucoside	581.3 ± 19.6	683.2 ± 28.1	496.4 ± 22.2	426.9 ± 12.9	590.7 ± 49.8	536.5 ± 16.9	0.443	0.663
Myricetin-3-*O*-glucuronide	16.0 ± 0.6a	38.5 ± 1.7b	32.9 ± 0.4b	19.1 ± 0.8a	39.6 ± 0.9b	40.6 ± 0.5b	0.001	0.281
*Cis*-kaempferol-3-*O*-glucoside	15.3 ± 1.5a	11.6 ± 1.4a	0.8 ± 0.1b	10.6 ± 2.3a	6.6 ± 0.2a	1.8 ± 0.2b	0.009	0.116
*Trans*-kaempferol-3-*O*-glucoside	74.3 ± 8.8a	57.7 ± 8.6a	2.2 ± 0.6b	49.7 ± 10.0a	27.9 ± 3.6a	6.1 ± 0.1b	0.000	0.007
Quercetin-3-*O*-glucoside	70.1 ± 3.3	70.0 ± 2.9	71.3 ± 12	68.4 ± 3.9	70.2 ± 1.1	69.6 ± 8.6	0.985	0.853
Quercetin-3-*O*-galactoside	34.0 ± 2.7a	25.6 ± 8.3a	2.5 ± 0.5b	34.5 ± 8.5a	19.2 ± 1.5ab	4.9 ± 2.8b	0.005	0.826
Quercetin-3-*O*-glucopyranoside	181.2 ± 6.4a	173.0 ± 35.2a	19.0 ± 3.5b	158.9 ± 34.8a	101.0 ± 7.7a	14.5 ± 0.5b	0.000	0.097
Quercetin-3-*O*-glucuronide	176.2 ± 16.0a	143.5 ± 28.0a	46.3 ± 6.3b	212.9 ± 50.1a	119.3 ± 2.1ab	53.2 ± 9.0b	0.000	0.687
Quercetin-3-*O*-rutinoside	10.5 ± 0.6a	6.1 ± 1.7b	1.5 ± 0.4c	9.3 ± 0.8a	5.2 ± 0.9ab	2.8 ± 0.5b	0.000	0.722
*Cis*-isorhamnetin-3-*O*-glucoside	95.7 ± 3.6a	79.2 ± 1.9b	98.2 ± 3.3a	76.0 ± 3.1a	85.3 ± 3.1a	91.8 ± 4.3a	0.012	0.033
*Trans*-isorhamnetin-3-*O*-glucoside	9.2 ± 0.2a	11.8 ± 2.2a	2.2 ± 0.8b	8.9 ± 0.1a	9.0 ± 0.5a	1.8 ± 0.2b	0.002	0.258
*Cis*-isorhamnetin-3-*O*-glucuronide	0.8 ± 0.1a	0.8 ± 0.2a	0.4 ± 0.1b	0.7 ± 0.1a	0.7 ± 0.1a	0.3 ± 0.1b	0.001	0.864
*Trans*-isorhamnetin-3-*O*-glucuronide	4.1 ± 0.6a	3.0 ± 1.7a	1.1 ± 0.4b	3.8 ± 0.8a	3.5 ± 0.9a	1.0 ± 0.5b	0.016	0.365
Syringetin-3-*O*-glucoside	15.7 ± 1.5	13.2 ± 1.1	17.4 ± 2.2	14.1 ± 1.8	14.0 ± 1.6	16.4 ± 2.4	0.774	0.583
**Anthocyanins (mg · g**^**-1**^**FW)**								
Malvidin-3-*O*-glucoside	28.0 ± 3.9	28.5 ± 2.1	34.5 ± 2.0	31.8 ± 0.8	32.1 ± 2.1	32.1 ± 5.5	0.477	0.509
Petunidin-3-*O*-glucoside	12.5 ± 1.2	11.6 ± 1.1	12.8 ± 1.4	13.3 ± 0.3	12.4 ± 0.9	11.5 ± 1.4	0.655	0.902
Delphinidin-3-*O*-glucoside	11.2 ± 0.7	9.7 ± 1.0	10.4 ± 1.5	11.4 ± 0.3	10.4 ± 0.8	9.2 ± 1.0	0.237	0.912
Peonidin-3-*O*-glucoside	5.2 ± 0.3	5.4 ± 1.0	3.8 ± 0.1	5.6 ± 0.4	6.3 ± 0.7	5.5 ± 0.7	0.883	0.843
Cyanidin-3-*O*-glucoside	2.9 ± 0.2	2.7 ± 0.7	3.3 ± 1.8	2.5 ± 0.3	3.0 ± 0.4	2.7 ± 0.9	0.922	0.729
Malvidin-3-*O*-(6´-acetyl) glucoside	4.9 ± 0.6	5.3 ± 0.4	5.3 ± 0.5	4.6 ± 0.2	4.4 ± 0.5	4.4 ± 1.0	0.983	0.113
Petunidin-3-*O*-(6´-acetyl) glucoside	0.2 ± 0.0a	0.2 ± 0.0a	0.1 ± 0.0a	0.2 ± 0.0a	0.1 ± 0.0a	0.1 ± 0.0b	0.001	0.001
Delphinidin-3-*O*-(6´-acetyl) glucoside	0.5 ± 0.1	0.5 ± 0.0	0.5 ± 0.0	0.5 ± 0.0	0.5 ± 0.0	0.5 ± 0.1	0.929	0.424
Peonidin-3-*O*-(6´-acetyl) glucoside	1.2 ± 0.2	1.4 ± 0.1	1.6 ± 0.1	1.3 ± 0.0	1.5 ± 0.1	1.5 ± 0.3	0.126	0.940
Cyanidin-3-*O*-(6´-acetyl) glucoside	0.4 ± 0.1	0.4 ± 0.0	0.4 ± 0.0	0.4 ± 0.0	0.4 ± 0.0	0.4 ± 0.1	0.910	0.360
Malvidin-3-*O*-(6´-*p*-coumaroyl) glucoside	12.2 ± 1.8	14.6 ± 0.6	16.3 ± 1.7	12.1 ± 0.3	14.0 ± 0.8	14.3 ± 3.3	0.168	0.538
Petunidin-3-*O*-(6´-*p*-coumaroyl) glucoside	3.8 ± 0.5	4.3 ± 0.3	4.5 ± 0.4	3.9 ± 0.1	3.8 ± 0.3	4.0 ± 0.8	0.720	0.368
Delphinidin-3-*O*-(6´-*p*-coumaroyl) glucoside	0.2 ± 0.0a	0.2 ± 0.0a	0.1 ± 0.0a	0.1 ± 0.0a	0.1 ± 0.0a	0.1 ± 0.0b	0.000	0.001
Cyanidin-3-*O*-(6´-*p*-coumaroyl) glucoside	0.8 ± 0.1	0.8 ± 0.1	0.8 ± 0.1	0.8 ± 0.0	0.8 ± 0.1	0.7 ± 0.0	0.586	0.196

MSPC and MIPC, bulk levels of methanol-soluble and -insoluble phenolic compounds (as the area under the absorbance curve in the interval 280–400 nm of the absorbance spectrum per mg FW). All the individual compounds were found in the methanol-soluble fraction except *p*-coumaric and syringic acids. Different letters mean significant differences between treatments for each ripeness level. Means ± SE are shown. Significance values in ANOVA for the differences in radiation treatments and berry saccharimetric level are shown (*P*-rad and *P*-s, respectively).

The degree of berry ripening only influenced significantly the concentrations of 11 (out of 41) compounds analysed, and eight of them (including four out of five analyzed flavanols) significantly decreased with increased berry density (Table 1). Caffeoyl-tartaric and *p*-coumaric acids were the only compounds that increased with ripeness. Thus, a higher number of phenolic compounds in Tempranillo berry skin was altered by solar UV radiation than by the ripening degree. In summary, flavonols increased with UV radiation while flavanols decreased concurrently to TSS gain.

### Solar UV-mediated gene expression in Tempranillo berry skin

In view that solar UV radiation had a major influence on the skin phenolic composition of Tempranillo berries reaching maturity, transcriptome was analysed in the same samples to search for putatively related changes in gene expression as well as other independent transcriptional effects of UV radiation. The whole normalized microarray expression dataset (Additional file 1) was firstly analysed in a PCA that revealed a more limited effect of UV radiation on gene expression than that of berry density (7% and 19% of total variance in gene expression, respectively). Furthermore, a stronger effect of solar UV radiation on the transcriptome of 26 ºBrix berries when compared to that in 23 ºBrix berries was patent on this plot (Additional file 3).

**Figure 4:**
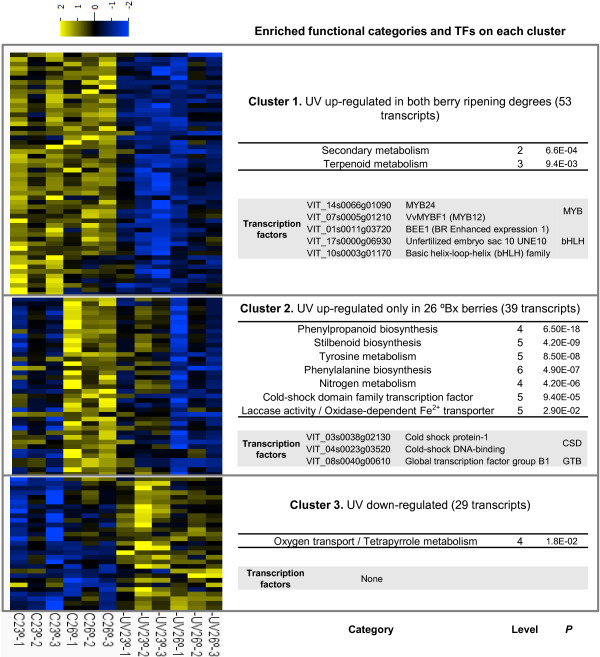
**Expression and functional analysis of UV-differentially expressed genes in Tempranillo berry skin.** Expression heat-map of UV-differentially expressed genes (*P* <0.01 and |Fold change| ≥2 at least for one berry density) grouped according to a three k-means clustering. Row normalized Log_2_ expression is represented for each sample. The number of transcripts, a summary of their significantly enriched functional categories (Benjamini-Hochberg adjusted *P* <0.05) and in a grey box, the transcription factors included are indicated in the right side of each cluster.

Next, the effect of UV radiation and its interaction with the harvested grape ripening stages were specifically analysed searching for significantly DE transcripts in a two-factor ANOVA (*P* ≤0.01 and fold change ≥2). Accordingly to PCA results, 122 UV-DE transcripts were identified when compared to 157 density-DE transcripts (Additional files 4 and 5). UV-DE transcripts were further characterized by grouping them according to their expression profiles in the two berry ripening degrees under both analysed UV radiation conditions. Three clusters were generated in a k-means analysis as the optimum number of clusters assessed in a Gap analysis (Additional file 6). Cluster 1 included 53 transcripts up-regulated by UV radiation independently of the berry density; cluster 2 included 39 transcripts up-regulated by UV radiation only in the skin of 26 ºBrix berries; and cluster 3 consisted of 29 UV radiation down-regulated transcripts, which mainly affected 23 ºBrix berries (Figure 4 and Additional file 4). All three expression profiles were analysed for functional enrichment. Cluster 1 was enriched in secondary metabolism and terpenoid metabolism pathway transcripts; while cluster 2 was enriched in phenylpropanoid and stilbenoid biosynthetic pathways. Cluster 2 was also enriched in metabolic pathways leading to phenylpropanoid precursors, i.e., nitrogen metabolism, phenylalanine biosynthesis and tyrosine metabolism (Figure 4 and Additional file 7). The enrichment of the 'secondary metabolism’ category in cluster 1 was mainly participated by monoterpenoid biosynthetic genes (two linalool synthase [*VIT_00s0372g00060* and *VIT_00s0385g00020*], two 1,8-cineole synthase [*VIT_00s0271g00010* and *VIT_00s0266g00020*] and one geraniol 10-hydroxylase [*VIT_15s0048g01490*]), as well as by one flavonol synthase (*VIT_18s0001g03470* [*VvFLS1* = *FLS4*]), two flavonol glycosyltransferases *VvGT5* and *VvGT6* (*VIT_11s0052g01600* and *VIT_11s0052g01630*) and one sinapyl alcohol dehydrogenase (*VIT_18s0122g00450*) encoding transcripts. Two anthranilate benzoyltransferase (*VIT_03s0038g01330* and *VIT_11s0037g00570*) and one chorismate mutase (*VIT_01s0010g00480*) induced by UV radiation in both analysed berry densities could contribute to the biosynthesis of aromatic and phenolic precursors. Also in cluster 1, UV radiation up-regulated the expression of five transcription factors (TFs): three bHLH, *VvMYB24*, *VvMYBF1*; and one cytokinin-responsive *CGA1-like* (Figure 4 and Additional file 4). Alternatively, cluster 2 included six putative phenylalanine ammonia-lyase (PAL), six putative stilbene synthase (STS) and other putative phenylpropanoid biosynthetic transcripts such as one *p*-coumaroyl shikimate 3'-hydroxylase (*VIT_08s0040g00780*), one chalcone synthase (*VIT_16s0100g00860*), one cinnamate 4-hydroxylase (*VIT_11s0078g00290*) or one flavonoid 3-O-glucosyltransferase (*VIT_03s0017g02120*). Among regulatory genes, cluster 2 contained two cold-shock domain and one global transcription factor family TFs induced by UV radiation mainly in 26 ºBrix berries. UV radiation down-regulated transcripts (cluster 3) were only enriched in hemoglobin encoding transcripts and did not include any TF. Thus, these analyses identified that UV radiation activated secondary metabolism pathways leading to key precursors for grape and wine polyphenolic composition and flavour.

Concerning berry density-DE transcripts, 104 were up-regulated and 53 down-regulated in the skin of 26 ºBrix berries. The 'Oxidative stress response’ was enriched among 26 ºBrix up-regulated transcripts (Additional file 7). Several laccase, one peroxidase, one dehydroascorbate reductase and one glutathione S-transferase encoding transcripts up-regulated in 26 ºBrix berries (Additional file 5) determined such enrichment. Relative to the phenylpropanoid metabolism, only one anthocyanidin reductase and one flavonoid 3’,5’-hydroxilase encoding transcripts over-expressed in the skin of 26 ºBrix berries were density-DE (*VIT_00s0361g00040* and *VIT_08s0007g05160*, respectively). However, induction of both transcripts is opposed to the observed reduction of flavanols such as *cis*-epigallocatechin in the skin of 26° Brix berries (Table 1).

### UV signalling meta-analysis

A transcriptomic meta-analysis was carried out to check whether solar UV radiation could influence berry skin gene expression through the activation of the UV-B radiation-specific signalling pathway. Clusters of UV-DE transcripts identified in our experiment (Figure 4 and Additional file 4) were analysed for their possible enrichment in homologs to Arabidopsis genes induced by UV-B radiation in a UVR8-dependent manner [54]. The genes up-regulated by UV radiation in the berry skin independently of the berry ripening stage (cluster 1) were enriched in these homologs (Benjamini-Hochberg adjusted *P* = 1 · 10^-11^; Additional file 7); whereas cluster 2 and cluster 3 were not significantly enriched. The presence of eight homologs to Arabidopsis UVR8-dependent UV-B radiation-induced genes in cluster 1, including two photolyase (*VIT_04s0008g02670* and *VIT_09s0002g05990*) and flavonol biosynthetic *VvFLS1* and *VvGT5* transcripts (Additional file 4), determined such enrichment. In parallel, the grapevine reference genome was searched for homologs to Arabidopsis UV-B-signalling pathway genes (Table 2). One *UVR8* UV-B photoreceptor homolog was identified (*VvUVR8*). Grapevine homologs for other genes participating in the UV-B radiation signalling pathway were also found including two *ELONGATED HYPOCOTYL 5* (*HY5*) (*VvHY5-1* and *VvHY5-*2), two *CONSTITUTIVE PHOTOMORPHOGENIC 1* (*COP1*) (*VvCOP1-1* and *VvCOP1-2*) and one *REPRESSOR OF UV-B PHOTOMORPHOGENESIS 2* (*RUP2*) (*VvRUP*). Two different regions homologous to Arabidopsis RUP1 and RUP2 protein sequences were found in the VvRUP protein predicted in the 12X V1 annotation version of the grapevine reference genome; while grapevine ESTs database may indicate that actually, there are two different *RUP* homologs encoded within the *VvRUP* locus (data not shown). However, *VvRUP* was considered as a single gene for further analysis because the NimbleGen microarray probes were designed accordingly. *VvHY5-1*, *VvHY5-2* and *VvRUP* were significantly induced by UV radiation in the berry skin although this induction was only greater than two-fold for *VvRUP* (Table 2). These data suggest the presence of the UV-B signalling pathway in grapevine and its activation in the skin of Tempranillo berries exposed to solar UV radiation.

**Table 2:** Grapevine homologs to Arabidopsis UV-B signalling pathway proteins

**Protein**	**ID**	**Length (aa)**	**Query coverage (%)**	**Identity (%)**	**23 ºBrix (FUV+/FUV-)**^**a**^	**26 ºBrix (FUV+/FUV-)**^**a**^	***P***^**b**^
**UVR8** (AT5G63860, 440 aa)							
VvUVR8	VIT_07s0031g02560	445	98	82	0.03	0.07	-
**HY5** (AT5G11260, 168 aa)							
VvHY5-1	VIT_04s0008g05210	169	100	78	0.53	0.58	0.022
VvHY5-2	VIT_05s0020g01090	210	67	48	0.99	0.47	0.002
**COP1** (AT2G32950, 675 aa)							
VvCOP1-1	VIT_12s0059g01420	676	100	75	0.25	-0.26	-
VvCOP1-2	VIT_10s0523g00030	602	89	78	0.25	-0.07	-
**RUP1** (AT5G52250, 385 aa)							
VvRUP	VIT_16s0050g00020	770	94	60	1.49	1.36	0.004
**RUP2** (AT5G23730, 368 aa)							
VvRUP	VIT_16s0050g00020	770	98	64	1.49	1.36	0.004

Arabidopsis protein name abbreviations are highlighted in bold.

^a^Log_2_ of normalized expression ratio in the NimbleGen microarrays. ^b^Differential expression significance *P* <0.05 for UV factor in ANOVA.

## Discussion

The current study shows that solar UV radiation influences the grapevine berry skin transcriptome and phenolic composition at mid-altitudes where viticulture is most frequently practised. The environmental UV radiation levels to which the experimental plants were exposed along the study period were normal for the latitude and altitude of the experimental site [55]. Filter exclusion of solar UV-A and UV-B radiation limited the accumulation of several phenolic compounds in the skin of Tempranillo berries, which was correlated with alteration in the expression of a modest number of genes at harvest (0.4% of the grapevine transcriptome). At late ripening stages, solar UV radiation enhanced the expression of transcripts encoding enzymes involved in the biosynthesis of phenolic compounds, mainly flavonols, hydroxycinnamic acids and stilbenes, whose levels were increased by solar UV radiation. This inductive effect extended to transcripts encoding terpenoid biosynthetic enzymes in the pathway of other chief metabolites in grape and wine flavour traits. Furthermore, these effects took place without reducing berry growth or hastening saccharimetric ripening, which contrasted with the berry growth reduction and saccharimetry alteration generated by the UV radiation received by Malbec grapes at high-altitudes (1500 m asl [33]). These results, together with previous studies on vine physiology, suggest that grapevine is adapted to the current environmental UV levels that are not harmful for its physiology; rather they increase its tolerance to stress conditions and result beneficial for improving fruit composition [3,4,9].

### Promotion of secondary metabolism in the grapevine berry skin by solar UV

Increased accumulation of glycosylated non-trisubstituted flavonols was the most evident impact of solar UV radiation on the skin phenolic composition of Tempranillo berries, with minor effects on the accumulation of anthocyanins and flavanols, as described for other grapevine cultivars [6,10,11,27]. Although genotype-dependent responses cannot be discarded, higher UV radiation doses could be required for increasing anthocyanins knowing that Malbec grapes only responded to UV radiation in this manner at high-altitudes [7]. However, an effect of higher temperatures at low altitude, which inhibit anthocyanin accumulation, cannot be discarded from that experiment [7,56,57].

Under the current study’s conditions, UV-A and UV-B radiation effects cannot be distinguished; however, a higher inductive effect of UV-B radiation on flavonols accumulation has been shown in previous studies despite they hold higher UV-A than UV-B radiation-screening capacity [27]. Indeed, flavonols have been suggested as the flavonoids most probably involved in antioxidative activities *in planta* and, thus, additional functions to UV radiation-screeners might be important for the photoprotection and the cross-tolerance conferred by these UV radiation-responsive compounds [16,25,58]. Other hints of antioxidant systems activation were not observed among UV radiation-responsive genes in our experiment. In contrast, the induction of two photolyase-encoding genes indicated the activation of photoprotective and repair mechanisms additionally to the presumably radiation-screening activity of UV radiation-increased *p*-coumaric acid and flavonols.

Consistently with alteration of berry skin composition, flavonoid-related transcripts induced by UV radiation independently of the berry ripening stage were mainly concerned with the pathway of flavonol biosynthesis. *VvFLS1* was amongst the most greatly induced transcripts in our experiments and, in fact, it encodes the flavonol synthase that is developmentally expressed during berry ripening [31] and the only one induced by UV radiation in Cabernet Sauvignon berries among several flavonoid biosynthetic genes analysed by Koyama et al. [11]. Induction of *VvFLS1* correlating with increased flavonols accumulation in the berry in response to sunlight radiation has been described elsewhere [11,29,30,59]. Flavonol glycosyltransferases *VvGT5* and *VvGT6* were concurrently induced by UV radiation and this mechanism could be important for the accumulation of glycosylated flavonols, which are non-toxic for plant cells [60,61]. The tendency for the accumulation of flavonol glycosylated structures other than the glucosylated ones might be in agreement with substrate preferences of VvGT5 and VvGT6 for UDP-glucuronic acid and UDP-glucose/UDP-galactose, respectively [60]. Considering that flavonol levels seem to be mainly affected by the UV spectrum of sunlight [11,27], an increased dose of UV radiation might mediate the promotion of *VvFLS1* expression and non-trisubstituted flavonols accumulation induced by viticultural practices such as defoliation [62]. In this line, berry skin flavonols are also increased in early defoliated Tempranillo vines grown at mid-altitude [63]. However, an accurate balance on canopy control by defoliation is necessary to avoid negative secondary consequences of these practices on grape development and composition [6]. Pruning and trellis systems allowing for the reception of a high solar irradiance but at the same time maintaining a suitable canopy for an effective photosynthesis may involve an interesting alternative to defoliation [64]. For instance, foldable trellis systems might provide a useful strategy to control the incident irradiation and the exposed canopy depending on the environmental conditions and the grape composition requirements [65].

The content of *p*-coumaric acid, a hydroxycinnamic acid with striking UV radiation absorption and antioxidant capacities, was also increased in the skin of UV radiation-exposed Tempranillo berries. Its accumulation could result from the hydrolysis of coumaroyl-tartaric acid, which decreased in the presence of solar UV radiation. PAL and cinnamate 4-hydroxylase encoding transcripts induced by UV radiation in 26° Brix berries could also contribute to enhance the biosynthesis of hydroxycinnamic acids. Interestingly, this UV radiation response might be specific of Tempranillo berries in view that hydroxycinnamic acids levels were not altered by UV radiation exposure in other grapevine cultivars [27,33]. Alternatively, the specific measurement of cell wall individual phenolic compounds (MIPC) in the present study could have led to this finding.

Stilbenes concentration and related gene expression was also altered by the imposed UV radiation treatments. Although resveratrol and the sum of stilbenes in both analysed berry densities were greater under FUV+ than in Ambient and FUV- treatments, higher expression of *PAL* and *STS* stilbenoid biosynthetic genes under FUV+ treatment was only detected in 26 ºBrix berries (Figures 3 and 4, Table 1 and Additional file 4). This result suggests that causes other than gene expression differences identified at the sampling time could underlie the effect of UV radiation on stilbene levels in 23 ºBrix berries. Co-induction of *PAL* and *STS* transcripts during berry ripening and in response to UV-B radiation in grapevine leaves has also been described before [66-68]. Induction of these genes by UV radiation specifically in 26 ºBrix berries might be related to berry dehydration bearing in mind that at late ripening stages berry sugar concentration mainly increases by dehydration; whereas water stress and postharvest berry wilting enhance stilbene biosynthesis and accumulation [69,70]. Indeed, UV irradiation of berries has proved to intensify the basal production of resveratrol in the berry skin during postharvest berry storage [71]. Moreover, only solar UV radiation doses present at high-altitudes have been shown to increase resveratrol accumulation in Malbec berry skin [7]. Thus, the lower UV irradiance concerning our experimental conditions could interact with the higher berry temperatures measured under the FUV+ filter to enhance stilbenes production. In fact, high temperatures have been shown to induce *STS* expression and stilbenes accumulation in the berry skin, mainly at late ripening stages or during postharvest wilting [70,72].

Monoterpene biosynthetic enzyme encoding transcripts, including a 1,8-cineole/eucalyptol synthase and two linalool synthases, were induced by solar UV radiation in the skin of berries from both tested ripening stages. Similar expression changes could cause the UV-B radiation-promoted increase of terpenoids such as eucalyptol in the skin of Malbec berries [8]. As the metabolites produced by the up-regulated transcripts-encoded enzymes (i.e.: eucalyptol and linalool) are sources of appreciated aromatic perceptions from red and white wines [73-75], it would be interesting to study whether higher solar UV radiation during berry ripening enhances the accumulation of these aromas in Tempranillo wines.

In addition to UV radiation effects, the experimental set up also allowed for identifying a differential accumulation of myricetin, myricetin-3-*O*-glucuronide and *cis*-isorhamnetil-3-*O*-glucoside between filter-involving treatments and the environmental situation. Irradiation-independent factors such as higher temperature or the lack of wind-related effects under the polymetacrylate screens are speculated to underlie these differences. In fact, photosynthesis was higher under both filters than under the Ambient treatment (data not shown). These factors could promote the glucuronidation of myricetin considering that under both filter screens the reduction in aglycon levels correlated with the increase in the glucuronidated form. However, detection of myrcetin aglycon resulting from hydrolysis during the extraction procedures cannot be discarded since glycosylated flavonols usually accumulate in grapes.

### Signalling cascade activated by solar UV in the grape skin

Components of the UV-B perception and signalling pathway were identified in the grapevine reference genome including a putative UV-B radiation photoreceptor, *VvUVR8*, whose expression was not induced by UV radiation. Similarly, the expression of the Arabidopsis *UVR8* homolog is not UV radiation-inducible and, instead, its encoded protein interacts with UV radiation to initiate the UV-B signalling cascade [76,77]. The interaction between UVR8 and COP1 play a central role in the UV-B signalling pathway according to Arabidopsis studies [78,79]. We also identified two *COP1* homologs in the grapevine genome and, indeed, in spite of phylogenetic divergences and organ function disparities between the grapevine ripe berry skin and Arabidopsis leaves, as well as possible responses to UV-A radiation under our experimental conditions, transcripts induced by solar UV radiation in the skin of both analysed berry ripening degrees were enriched in Arabidopsis UVR8-dependent UV-B radiation-induced homologs. These results suggest that UV-B radiation could trigger responses in the grapevine berry skin through the *VvUVR8* UV-B photoreceptor homolog. In this manner, *VvUVR8* might mediate the UV-B induced accumulation of flavonols in the grape skin by up-regulating *VvFLS1* and *VvGT5* as described for their Arabidopsis flavonols biosynthetic gene homologs [24]. *VvGT6* might also be under similar control given that this gene probably appeared as a duplication of *VvGT5*[60]. Also induced by UV radiation in Tempranillo berry skin was *VvMYBF1*, which is a sunlight-induced TF directly promoting *VvFLS1* expression and flavonol biosynthesis [12,29]. Thus, VvMYBF1 could act downstream of VvUVR8 in the UV-B signalling cascade taking into account that UVR8 binds to the promoter of its Arabidopsis homolog *MYB12*[80].

UVR8 is necessary for the UV-B-induced *HY5* over-expression in Arabidopsis [54]; whereas *VvHY5-1* and *VvHY5-2* grapevine homologs were modestly up-regulated by solar UV radiation in the grape skin. *VvRUP* was also up-regulated. It is a homologous gene to *AtRUP1* and *AtRUP2*, which in Arabidopsis are induced by UV-B radiation in a UVR8-dependent manner and code for repressors of the UV-B signalling pathway [81]. These coincidences may suggest that a similar feedback loop to that identified in Arabidopsis [78] could tune this pathway in grapevine.

Although the inflorescence/berry specific *VvMYB24* TF is less characterized than *VvMYBF1*[82], it was even more up-regulated by UV radiation in Tempranillo berry skin than *VvMYBF1*. Thus, it could be interesting to check whether *VvMYB24* could play a role in the UV stimulation of secondary metabolism in grapevine berries. A similar role could be expected for all three bHLH transcripts that showed the same expression profile. All these TFs might be good candidates to regulate monoterpene synthases induced by UV radiation, similarly as VvMYBF1 does with flavonol biosynthetic genes. Since regulation of terpenoid biosynthesis remains largely unknown, it would be worth studying whether any of these TFs control this pathway. On the other hand, UV radiation-induced expression of gibberellin 2-oxidase (*VIT_10s0116g00410*) and GCA1-like TF encoding transcripts might suggest that UV radiation represses gibberellin signalling in the grape skin [83].

## Conclusions

Solar UV radiation levels reaching the Earth’s surface in the common altitudes used for grapevine growing influence grape berry skin gene expression and phenolic composition. Indeed, rather than activation of stress responses, solar UV radiation seems to trigger regulatory responses through the plant UV-B signalling cascade in grapevine berries, which results in the activation of phenylpropanoids and terpenoids biosynthesis together with other protective responses. These results contribute to our understanding of the impact of UV radiation on grapevine berry ripening. They may serve of value for decision-making on viticultural practices given that environmental UV radiation activated metabolic pathways rendering accumulation of compounds, which improve grape features for winemaking purposes, in the absence of other negative responses in the berry skin under mid-altitude and specific climate environment. Nonetheless, it should be confirmed whether UV radiation exerts similar control on berry skin gene expression and metabolism in different ripening stages, genotypes and environments. Finally, transcription factors that are up-regulated by UV such as *VvMYBF1*, *VvMYB24* or several *bHLH* could be good candidate genes for reverse genetics to check on their role in the control of UV radiation-activated metabolic pathways. Once confirmed, they could be targets for genetic selection useful in breeding programs aimed at improving grape features.

### Availability of supporting data

The full microarray expression data are available on GEO database under the accession number GSE54636.

## Abbreviations

Ambient: No filter control treatment; ANOVA: Analysis of variance; Asl: Above sea level; COP1: CONSTITUTIVE PHOTOMORPHOGENIC 1; DE: Differentially expressed; FLS: Flavonol synthase; GT: Glycosyl transferase; FUV-: UV-blocking filter treatment; FUV+: UV-transmitting filter treatment; FW: Fresh weight; GEO: Gene Expression Ominibus; HPLC: High-performance liquid chromatography; HPLC-MS: High-performance liquid chromatography-mass spectrometry; HY5: ELONGATED HYPOCOTYL 5; MIPC: Bulk level of methanol-insoluble phenolic compounds; MSPC: Bulk level of methanol-soluble phenolic compounds; PAL: Phenylalanine ammonia-lyase; PAR: Photosynthetically active radiation; PCA: Principal component analysis; RUP: Repressor of UV-B 3photomorphogenesis; STS: Stilbene synthase; TSS: Total soluble solids; UPLC: Ultra-performance liquid chromatography; UV: UV radiation; UV_BE_: Biologically effective UV radiation; UVR8: UVB-RESISTANCE 8; VSP: Vertical shoot positioning.

## Competing interests

The authors declare that they have no competing interests.

## Authors’ contributions

PCB participated in the design of experiments, berry sampling and classification, analysis of data and drafted the manuscript. MPD, JMA and ENO carried out experiments and analysed data. ENO, JT, MPD and JMMZ conceived the study and participated in the design of experiments. ENO and MPD participated in manuscript drafting. All authors critically revised the manuscript. All authors read and approved the final manuscript.

## Supplementary Material

Additional file 1 RMA normalized gene expression dataset. Click here for file

Additional file 2 Effects of radiation treatments and berry ripening degree on berry weight and skin to berry ratio. Click here for file

Additional file 3 PCA of RMA normalized gene expression. Click here for file

Additional file 4 UV-DE transcripts and clustering. Click here for file

Additional file 5 Density-DE transcripts. Click here for file

Additional file 6 Gap statististic analysis of UV-DE transcripts. Click here for file

Additional file 7 Functional enrichment analysis of DE transcripts. Click here for file

## References

[B1] Hollósy F Effects of ultraviolet radiation on plant cells Micron 2002 33 179 197 11567887 10.1016/s0968-4328(01)00011-7

[B2] McKenzie RL Aucamp PJ Bais AF Bjorn LO Ilyas M Changes in biologically-active ultraviolet radiation reaching the Earth’s surface Photochem Photobiol Sci 2007 6 218 231 17344959 10.1039/b700017k

[B3] Jug T Rusjan D Advantages and disadvantages of UV-B radiations on grapevine (*Vitis* sp.) Emirates J Food Agric 2012 24 576 585

[B4] Núñez-Olivera E Martínez-Abaigar J Tomás R Otero S Arróniz-Crespo M Physiological effects of Solar Ultraviolet-B exclusion on two cultivars of Vitis vinifera L. from La Rioja, Spain Am J Enology Viticulture 2006 57 441 448

[B5] Martinez-Luscher J Morales F Delrot S Sanchez-Diaz M Gomes E Aguirreolea J Pascual I Short- and long-term physiological responses of grapevine leaves to UV-B radiation Plant Sci 2013 213 114 122 24157214 10.1016/j.plantsci.2013.08.010

[B6] Gregan SM Wargent JJ Liu L Shinkle J Hofmann R Winefield C Trought M Jordan B Effects of solar ultraviolet radiation and canopy manipulation on the biochemical composition of Sauvignon Blanc grapes Aust J Grape Wine Res 2012 18 227 238

[B7] Berli F D’Angelo J Cavagnaro B Bottini R Wuilloud R Silva MF Phenolic composition in grape (Vitis vinifera L. cv. Malbec) ripened with different solar UV-B radiation levels by capillary zone electrophoresis J Agric Food Chem 2008 56 2892 2898 18412357 10.1021/jf073421+

[B8] Gil M Bottini R Berli F Pontin M Silva MF Piccoli P Volatile organic compounds characterized from grapevine (Vitis vinifera L. cv. Malbec) berries increase at pre-harvest and in response to UV-B radiation Phytochemistry 2013 96 148 157 24075072 10.1016/j.phytochem.2013.08.011

[B9] Kuhn N Guan L Dai ZW Wu BH Lauvergeat V Gomes E Li SH Godoy F Arce-Johnson P Delrot S Berry ripening: recently heard through the grapevine J Exp Bot In press. First published online November 27, 2013 doi:10.1093/jxb/ert395. 10.1093/jxb/ert395 24285825

[B10] Spayd SE Tarara JM Mee DL Ferguson JC Separation of sunlight and temperature effects on the composition of Vitis vinifera cv Merlot Berries Am J Enology Viticulture 2002 53 171 182

[B11] Koyama K Ikeda H Poudel PR Goto-Yamamoto N Light quality affects flavonoid biosynthesis in young berries of Cabernet Sauvignon grape Phytochemistry 2012 78 54 64 22455871 10.1016/j.phytochem.2012.02.026

[B12] Czemmel S Stracke R Weisshaar B Cordon N Harris NN Walker AR Robinson SP Bogs J The grapevine R2R3-MYB transcription factor VvMYBF1 regulates flavonol synthesis in developing grape berries Plant Physiol 2009 151 1513 1530 19741049 10.1104/pp.109.142059 PMC2773091

[B13] Kolb CA Kaser MA Kopecky J Zotz G Riederer M Pfundel EE Effects of natural intensities of visible and ultraviolet radiation on epidermal ultraviolet screening and photosynthesis in grape leaves Plant Physiol 2001 127 863 875 11706169 PMC129258

[B14] Tuteja N Ahmad P Panda BB Tuteja R Genotoxic stress in plants: shedding light on DNA damage, repair and DNA repair helicases Mutat Res 2009 681 134 149 18652913 10.1016/j.mrrev.2008.06.004

[B15] Blokhina O Virolainen E Fagerstedt KV Antioxidants, oxidative damage and oxygen deprivation stress: a review Ann Bot 2003 91 179 194 12509339 10.1093/aob/mcf118 PMC4244988

[B16] Agati G Tattini M Multiple functional roles of flavonoids in photoprotection New phytol 2010 186 786 793 20569414 10.1111/j.1469-8137.2010.03269.x

[B17] Berli FJ Moreno D Piccoli P Hespanhol-Viana L Silva MF Bressan-Smith R Cavagnaro JB Bottini R Abscisic acid is involved in the response of grape (Vitis vinifera L.) cv. Malbec leaf tissues to ultraviolet-B radiation by enhancing ultraviolet-absorbing compounds, antioxidant enzymes and membrane sterols Plant Cell Environ 2010 33 1 10 19781012 10.1111/j.1365-3040.2009.02044.x

[B18] Hideg E Jansen MA Strid A UV-B exposure, ROS, and stress: inseparable companions or loosely linked associates? Trends Plant Sci 2013 18 107 115 23084465 10.1016/j.tplants.2012.09.003

[B19] Jansen MA Bornman JF UV-B radiation: from generic stressor to specific regulator Physiol Plant 2012 145 501 504 22646504 10.1111/j.1399-3054.2012.01656.x

[B20] Casal JJ Photoreceptor signaling networks in plant responses to shade Annu Rev Plant Biol 2013 64 403 427 23373700 10.1146/annurev-arplant-050312-120221

[B21] Bassman JH Ecosystem consequences of enhanced solar ultraviolet radiation: secondary plant metabolites as mediators of multiple trophic interactions in terrestrial plant communities Photochem Photobiol 2004 79 382 398 15191046 10.1562/si-03-24.1

[B22] Jansen MA Coffey AM Prinsen E UV-B induced morphogenesis: four players or a quartet? Plant Signal Behav 2012 7 1185 1187 22899069 10.4161/psb.21260 PMC3489657

[B23] Tilbrook K Arongaus AB Binkert M Heijde M Yin R Ulm R The UVR8 UV-B Photoreceptor: perception signaling and response Arabidopsis Book 2013 11 e0164 23864838 10.1199/tab.0164 PMC3711356

[B24] Morales LO Brosche M Vainonen J Jenkins GI Wargent JJ Sipari N Strid A Lindfors AV Tegelberg R Aphalo PJ Multiple roles for UV RESISTANCE LOCUS8 in regulating gene expression and metabolite accumulation in Arabidopsis under solar ultraviolet radiation Plant Physiol 2013 161 744 759 23250626 10.1104/pp.112.211375 PMC3561016

[B25] Agati G Brunetti C Di Ferdinando M Ferrini F Pollastri S Tattini M Functional roles of flavonoids in photoprotection: new evidence, lessons from the past Plant Physiol Biochem 2013 72 35 45 23583204 10.1016/j.plaphy.2013.03.014

[B26] Adams DO Phenolics and ripening in Grape Berries Am J Enology Viticulture 2006 57 249 256

[B27] Kolb CA Kopecký J Riederer M Pfündel EE UV screening by phenolics in berries of grapevine (*Vitis vinifera*) Funct Plant Biol 2003 30 1177 1186 10.1071/FP03076 32689099

[B28] Lutz M Jorquera K Cancino B Ruby R Henriquez C Phenolics and antioxidant capacity of table grape (Vitis vinifera L.) cultivars grown in Chile J Food Sci 2011 76 C1088 C1093 21819404 10.1111/j.1750-3841.2011.02298.x

[B29] Matus JT Loyola R Vega A Pena-Neira A Bordeu E Arce-Johnson P Alcalde JA Post-veraison sunlight exposure induces MYB-mediated transcriptional regulation of anthocyanin and flavonol synthesis in berry skins of Vitis vinifera J Exp Bot 2009 60 853 867 19129169 10.1093/jxb/ern336 PMC2652055

[B30] Downey MO Harvey JS Robinson SP The effect of bunch shading on berry development and flavonoid accumulation in Shiraz grapes Aust J Grape Wine Res 2004 10 55 73

[B31] Downey MO Harvey JS Robinson SP Synthesis of flavonols and expression of flavonol synthase genes in the developing grape berries of Shiraz and Chardonnay (Vitis vinifera L.) Aust J Grape Wine Res 2003 9 110 121

[B32] Zhang ZZ Che XN Pan QH Li XX Duan CQ Transcriptional activation of flavan-3-ols biosynthesis in grape berries by UV irradiation depending on developmental stage Plant Sci 2013 208 64 74 23683931 10.1016/j.plantsci.2013.03.013

[B33] Berli FJ Fanzone M Piccoli P Bottini R Solar UV-B and ABA are involved in phenol metabolism of Vitis vinifera L. increasing biosynthesis of berry skin polyphenols J Agric Food Chem 2011 59 4874 4884 21469737 10.1021/jf200040z

[B34] Zhang ZZ Li XX Chu YN Zhang MX Wen YQ Duan CQ Pan QH Three types of ultraviolet irradiation differentially promote expression of shikimate pathway genes and production of anthocyanins in grape berries Plant Physiol Biochem 2012 57 74 83 22683531 10.1016/j.plaphy.2012.05.005

[B35] Kataoka I Sugiyama A Beppu K Role of Ultraviolet Radiation in accumulation of Anthocyanin in Berries of 'Gros Colman’ Grapes (Vitis vinifera L.) J Jpn Soc Horticultural Sci 2003 72 1 6

[B36] Woodall GS Stewart GR Do anthocyanins play a role in UV protection of the red juvenile leaves of Syzygium? J Exp Bot 1998 49 1447 1450

[B37] da Silva PF Paulo L Barbafina A Eisei F Quina FH Macanita AL Photoprotection and the photophysics of acylated anthocyanins Chemistry 2012 18 3736 3744 22334328 10.1002/chem.201102247

[B38] Flint SD Caldwell MM A biological spectral weighting function for ozone depletion research with higher plants Physiol Plant 2003 117 137 144

[B39] Pou A Diago MP Medrano H Baluja J Tardaguila J Validation of thermal indices for water status identification in grapevine Agric Water Manage 2014 134 60 72

[B40] Stein U Blaich R Wind R A novel method for non-destructive determination of the sugar content and for classification of grape berries Vitis 1983 22 15 22

[B41] Rolle L Segade SR Torchio F Giacosa S Cagnasso E Marengo F Gerbi V Influence of grape density and harvest date on changes in phenolic composition, phenol extractability indices, and instrumental texture properties during ripening J Agric Food Chem 2011 59 8796 8805 21749143 10.1021/jf201318x

[B42] Schnitzler J-P Jungblut TP Heller W Köfferlein M Hutzler P Heinzmann U Schmelzer E Ernst D Langebartels C Sandermann H Tissue localization of u.v.-B-screening pigments and of chalcone synthase mRNA in needles of Scots pine seedlings New Phytol 1996 132 247 258

[B43] Fabón G Martínez-Abaigar J Tomás R Núñez-Olivera E Effects of enhanced UV-B radiation on hydroxycinnamic acid derivatives extracted from different cell compartments in the aquatic liverwort Jungermannia exsertifolia subsp. cordifolia Physiol Plant 2010 140 269 279 20663084 10.1111/j.1399-3054.2010.01401.x

[B44] Gómez-Alonso S García-Romero E Hermosín-Gutiérrez I HPLC analysis of diverse grape and wine phenolics using direct injection and multidetection by DAD and fluorescence J Food Composition Anal 2007 20 618 626

[B45] Saenz-Navajas MP Ferreira V Dizy M Fernandez-Zurbano P Characterization of taste-active fractions in red wine combining HPLC fractionation, sensory analysis and ultra performance liquid chromatography coupled with mass spectrometry detection Anal Chim Acta 2010 673 151 159 20599029 10.1016/j.aca.2010.05.038

[B46] Reid KE Olsson N Schlosser J Peng F Lund ST An optimized grapevine RNA isolation procedure and statistical determination of reference genes for real-time RT-PCR during berry development BMC Plant Biol 2006 6 27 17105665 10.1186/1471-2229-6-27 PMC1654153

[B47] Irizarry RA Bolstad BM Collin F Cope LM Hobbs B Speed TP Summaries of Affymetrix GeneChip probe level data Nucleic Acids Res 2003 31 e15 12582260 10.1093/nar/gng015 PMC150247

[B48] Raychaudhuri S Stuart JM Altman RB Principal components analysis to summarize microarray experiments: application to sporulation time series Pac Symp Biocomput 2000 5 455 466 PMCID: PMC2669932 10902193 10.1142/9789814447331_0043 PMC2669932

[B49] Saeed AI Bhagabati NK Braisted JC Liang W Sharov V Howe EA Li J Thiagarajan M White JA Quackenbush J TM4 microarray software suite Methods Enzymol 2006 411 134 193 16939790 10.1016/S0076-6879(06)11009-5

[B50] Tibshirani R Walther G Hastie T Estimating the number of clusters in a data set via the gap statistic J Royal Stat Soc Ser B 2001 63 411 423

[B51] Medina I Carbonell J Pulido L Madeira SC Goetz S Conesa A Tarraga J Pascual-Montano A Nogales-Cadenas R Santoyo J García F Marbà M Montaner D Dopazo J Babelomics: an integrative platform for the analysis of transcriptomics, proteomics and genomic data with advanced functional profiling Nucleic Acids Res 2010 38 W210 W213 20478823 10.1093/nar/gkq388 PMC2896184

[B52] Grimplet J Van Hemert J Carbonell-Bejerano P Diaz-Riquelme J Dickerson J Fennell A Pezzotti M Martinez-Zapater JM Comparative analysis of grapevine whole-genome gene predictions, functional annotation, categorization and integration of the predicted gene sequences BMC Res Notes 2012 5 213 22554261 10.1186/1756-0500-5-213 PMC3419625

[B53] Al-Shahrour F Diaz-Uriarte R Dopazo J FatiGO: a web tool for finding significant associations of Gene Ontology terms with groups of genes Bioinformatics 2004 20 578 580 14990455 10.1093/bioinformatics/btg455

[B54] Brown BA Cloix C Jiang GH Kaiserli E Herzyk P Kliebenstein DJ Jenkins GI A UV-B-specific signaling component orchestrates plant UV protection Proc Natl Acad Sci U S A 2005 102 18225 18230 16330762 10.1073/pnas.0507187102 PMC1312397

[B55] Hader DP Lebert M Schuster M del Ciampo L Helbling EW McKenzie R ELDONET–a decade of monitoring solar radiation on five continents Photochem Photobiol 2007 83 1348 1357 18028208 10.1111/j.1751-1097.2007.00168.x

[B56] Carbonell-Bejerano P Santa Maria E Torres-Perez R Royo C Lijavetzky D Bravo G Aguirreolea J Sanchez-Diaz M Antolin MC Martinez-Zapater JM Thermotolerance Responses in Ripening Berries of Vitis vinifera L. cv Muscat Hamburg Plant Cell Physiol 2013 54 1200 1216 23659918 10.1093/pcp/pct071

[B57] Buttrose MS Hale CR Kliewer WM Effect of temperature on the composition of 'Cabernet Sauvignon’ Berries Am J Enology Viticulture 1971 22 71 75

[B58] Hernandez I Alegre L Van Breusegem F Munne-Bosch S How relevant are flavonoids as antioxidants in plants? Trends Plant Sci 2009 14 125 132 19230744 10.1016/j.tplants.2008.12.003

[B59] Fujita A Goto-Yamamoto N Aramaki I Hashizume K Organ-specific transcription of putative flavonol synthase genes of grapevine and effects of plant hormones and shading on flavonol biosynthesis in grape berry skins Biosci Biotechnol Biochem 2006 70 632 638 16556978 10.1271/bbb.70.632

[B60] Ono E Homma Y Horikawa M Kunikane-Doi S Imai H Takahashi S Kawai Y Ishiguro M Fukui Y Nakayama T Functional differentiation of the glycosyltransferases that contribute to the chemical diversity of bioactive flavonol glycosides in grapevines (Vitis vinifera) Plant Cell 2010 22 2856 2871 20693356 10.1105/tpc.110.074625 PMC2947185

[B61] Yin R Messner B Faus-Kessler T Hoffmann T Schwab W Hajirezaei MR von Saint Paul V Heller W Schaffner AR Feedback inhibition of the general phenylpropanoid and flavonol biosynthetic pathways upon a compromised flavonol-3-O-glycosylation J Exp Bot 2012 63 2465 2478 22249996 10.1093/jxb/err416 PMC3346215

[B62] Pastore C Zenoni S Fasoli M Pezzotti M Tornielli GB Filippetti I Selective defoliation affects plant growth, fruit transcriptional ripening program and flavonoid metabolism in grapevine BMC Plant Biol 2013 13 30 23433030 10.1186/1471-2229-13-30 PMC3599245

[B63] Diago MP Ayestaran B Guadalupe Z Poni S Tardaguila J Impact of Prebloom and fruit set basal leaf removal on the Flavonol and Anthocyanin composition of Tempranillo Grapes Am J Enology Viticulture 2012 63 367 376

[B64] Reynolds AG Vanden Heuvel JE Influence of Grapevine training systems on vine growth and fruit composition: a review Am J Enology Viticulture 2009 60 251 268

[B65] Carbonneau A Monte R Lopez F Ojeda H The foldable lyre: ecophysiological interest for management of light absorption and water; technological interest for mechanical harvesting J Int des Sci de la Vigne et du Vin 2004 38 89 95

[B66] Lijavetzky D Carbonell-Bejerano P Grimplet J Bravo G Flores P Fenoll J Hellín P Oliveros JC Martínez-Zapater JM Berry flesh and skin ripening features in Vitis vinifera as assessed by transcriptional profiling PLoS One 2012 7 e39547 22768087 10.1371/journal.pone.0039547 PMC3386993

[B67] Pontin MA Piccoli PN Francisco R Bottini R Martinez-Zapater JM Lijavetzky D Transcriptome changes in grapevine (Vitis vinifera L.) cv. Malbec leaves induced by ultraviolet-B radiation BMC Plant Biol 2010 10 224 20959019 10.1186/1471-2229-10-224 PMC3017828

[B68] Gatto P Vrhovsek U Muth J Segala C Romualdi C Fontana P Pruefer D Stefanini M Moser C Mattivi F Velasco R Ripening and genotype control stilbene accumulation in healthy grapes J Agric Food Chem 2008 56 11773 11785 19032022 10.1021/jf8017707

[B69] Deluc LG Decendit A Papastamoulis Y Merillon JM Cushman JC Cramer GR Water deficit increases stilbene metabolism in Cabernet Sauvignon berries J Agric Food Chem 2011 59 289 297 21128664 10.1021/jf1024888 PMC3015458

[B70] Versari A Parpinello GP Tornielli GB Ferrarini R Giulivo C Stilbene compounds and stilbene synthase expression during ripening, wilting, and UV treatment in Grape cv Corvina J Agric Food Chem 2001 49 5531 5536 11714355 10.1021/jf010672o

[B71] Cantos E Garcia-Viguera C de Pascual-Teresa S Tomas-Barberan FA Effect of Postharvest Ultraviolet Irradiation on Resveratrol and other Phenolics of Cv Napoleon Table Grapes JAgric Food Chem 2000 48 4606 4612 11052707 10.1021/jf0002948

[B72] Mori K Goto-Yamamoto N Kitayama M Hashizume K Loss of anthocyanins in red-wine grape under high temperature J Exp Bot 2007 58 1935 1945 17452755 10.1093/jxb/erm055

[B73] Farina L Boido E Carrau F Versini G Dellacassa E Terpene compounds as possible precursors of 1,8-cineole in red grapes and wines J Agric Food Chem 2005 53 1633 1636 15740051 10.1021/jf040332d

[B74] De Pinho PG Falque E Castro M Oliveira ESH Machado B Ferreira AC Further insights into the floral character of Touriga Nacional wines J Food Sci 2007 72 S396 S401 17995696 10.1111/j.1750-3841.2007.00405.x

[B75] Campo E Ferreira V Escudero A Cacho J Prediction of the wine sensory properties related to grape variety from dynamic-headspace gas chromatography-olfactometry data J Agric Food Chem 2005 53 5682 5690 15998133 10.1021/jf047870a

[B76] Kliebenstein DJ Lim JE Landry LG Last RL Arabidopsis UVR8 regulates ultraviolet-B signal transduction and tolerance and contains sequence similarity to human regulator of chromatin condensation 1 Plant Physiol 2002 130 234 243 12226503 10.1104/pp.005041 PMC166556

[B77] Rizzini L Favory JJ Cloix C Faggionato D O’Hara A Kaiserli E Baumeister R Schafer E Nagy F Jenkins GI Ulm R Perception of UV-B by the Arabidopsis UVR8 protein Science 2011 332 103 106 21454788 10.1126/science.1200660

[B78] Heijde M Ulm R Reversion of the Arabidopsis UV-B photoreceptor UVR8 to the homodimeric ground state Proc Natl Acad Sci U S A 2013 110 1113 1118 23277547 10.1073/pnas.1214237110 PMC3549095

[B79] Cloix C Kaiserli E Heilmann M Baxter KJ Brown BA O’Hara A Smith BO Christie JM Jenkins GI C-terminal region of the UV-B photoreceptor UVR8 initiates signaling through interaction with the COP1 protein Proc Natl Acad Sci U S A 2012 109 16366 16370 22988111 10.1073/pnas.1210898109 PMC3479605

[B80] Cloix C Jenkins GI Interaction of the Arabidopsis UV-B-specific signaling component UVR8 with chromatin Mol Plant 2008 1 118 128 20031919 10.1093/mp/ssm012

[B81] Gruber H Heijde M Heller W Albert A Seidlitz HK Ulm R Negative feedback regulation of UV-B-induced photomorphogenesis and stress acclimation in Arabidopsis Proc Natl Acad Sci U S A 2010 107 20132 20137 21041653 10.1073/pnas.0914532107 PMC2993346

[B82] Matus JT Aquea F Arce-Johnson P Analysis of the grape MYB R2R3 subfamily reveals expanded wine quality-related clades and conserved gene structure organization across Vitis and Arabidopsis genomes BMC Plant Biol 2008 8 83 18647406 10.1186/1471-2229-8-83 PMC2507771

[B83] Richter R Behringer C Muller IK Schwechheimer C The GATA-type transcription factors GNC and GNL/CGA1 repress gibberellin signaling downstream from DELLA proteins and PHYTOCHROME-INTERACTING FACTORS Genes Dev 2010 24 2093 2104 20844019 10.1101/gad.594910 PMC2939370

