# Supplementary material for: Solar ultraviolet radiation is necessary to enhance grapevine fruit ripening transcriptional and phenolic responses
Source: BMC Plant Biol. 2014 Jul 9;14:183. doi: 10.1186/1471-2229-14-183 (PMC4099137; doi:10.1186/1471-2229-14-183)
Supplement: Additional file 3 — PCA of RMA normalized gene expression. [file 1471-2229-14-183-S3.pdf]

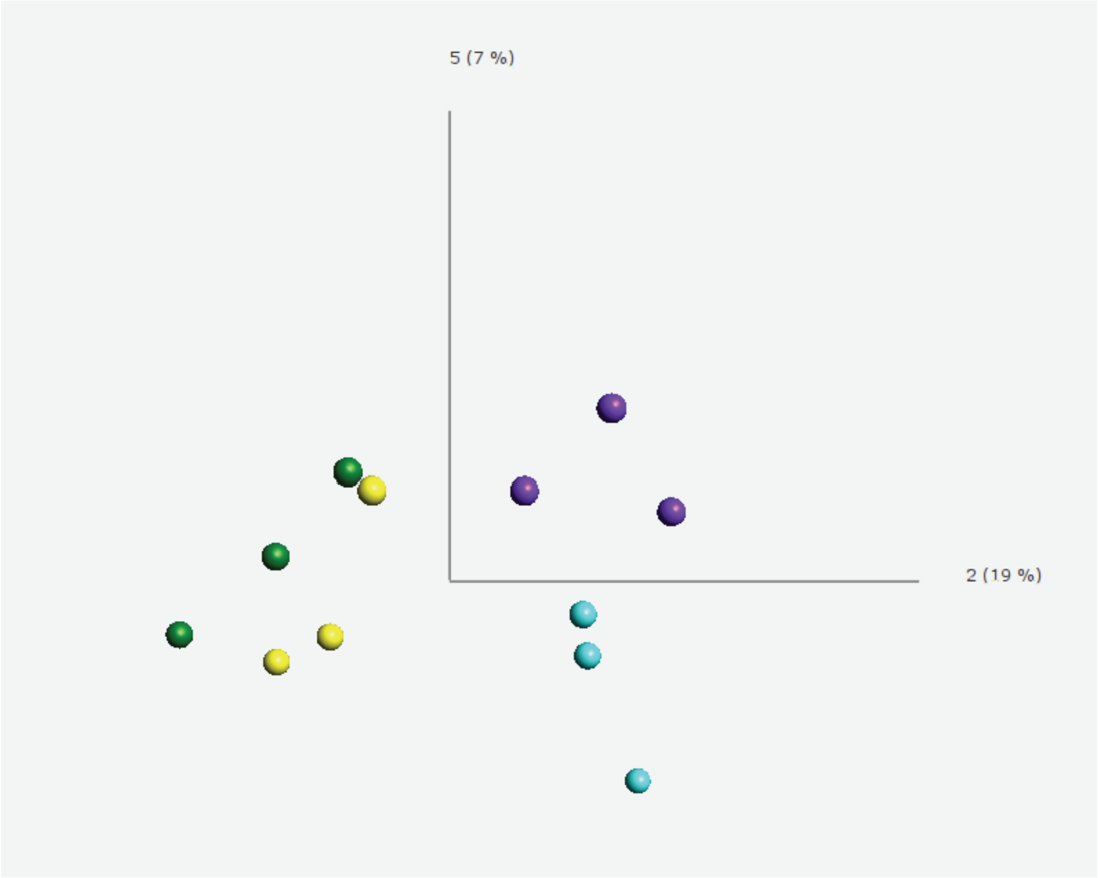

Figure S1. Principal component analysis of RMA normalized expression data in Tempranillo berry skin. Component 2 (explaining 19% of variance) and component 5 (7% of variance), reflecting berry density and radiation treatment effects, respectively, are shown. Green, UV-transmitting filter (FUV+) and 23 ºBrix; Yellow, UV-blocking filter and 23 ºBrix; Purple, FUV+ filter and 26 ºBrix; Blue, FUV- filter and 26 ºBrix.
