# Supplementary material for: Solar ultraviolet radiation is necessary to enhance grapevine fruit ripening transcriptional and phenolic responses
Source: BMC Plant Biol. 2014 Jul 9;14:183. doi: 10.1186/1471-2229-14-183 (PMC4099137; doi:10.1186/1471-2229-14-183)
Supplement: Additional file 6 — Gap statististic analysis of UV-DE transcripts. [file 1471-2229-14-183-S6.pdf]

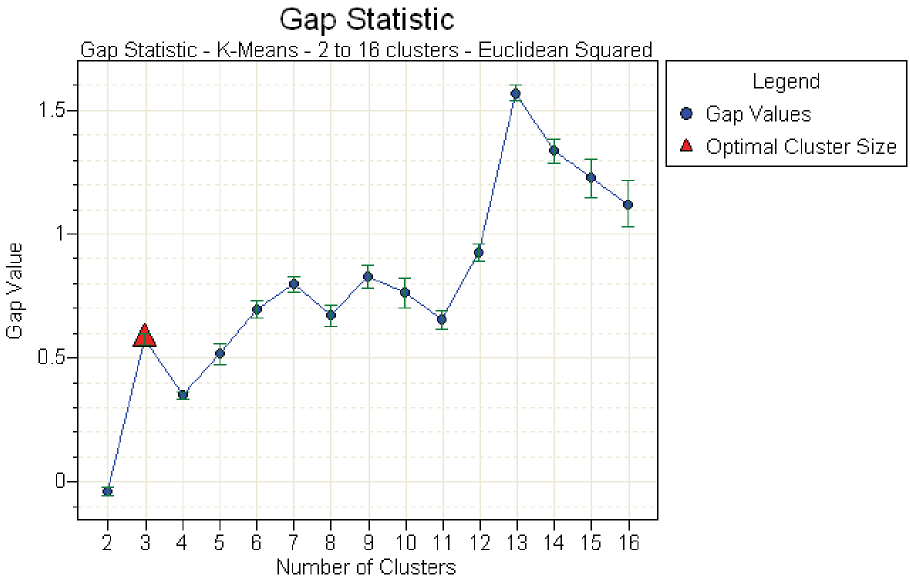

Figure S2. Gap statististic analysis of UV-differentially expressed genes in Tempranillo berry skin. The Gap score for each cluster number (between 2 and 16) using k-means clustering and Euclidean squared metrics is shown. The resulting optimum number of clusters is highlighted by a red triangle.
